# Acute Pulmonary Histoplasmosis Following COVID-19: Novel Laboratorial Methods Aiding Diagnosis

**DOI:** 10.3390/jof7050346

**Published:** 2021-04-28

**Authors:** Priscila Marques de Macedo, Andrea D’Ávila Freitas, Thiago Prudente Bártholo, Andrea Reis Bernardes-Engemann, Marcos de Abreu Almeida, Fernando Almeida-Silva, Rosely Maria Zancopé-Oliveira, Rodrigo Almeida-Paes

**Affiliations:** 1 Evandro Chagas National Institute of Infectious Diseases, Fiocruz, Rio de Janeiro 21040-900, Brazil; andrea.freitas@ini.fiocruz.br (A.D.F.); andrea.engemann@ini.fiocruz.br (A.R.B.-E.); marcos.almeida@ini.fiocruz.br (M.d.A.A.); fernando.almeida@ini.fiocruz.br (F.A.-S.); rosely.zancope@ini.fiocruz.br (R.M.Z.-O.); rodrigo.paes@ini.fiocruz.br (R.A.-P.); 2 Pulmonology and Tisiology Service, Pedro Ernesto University Hospital, University of the State of Rio de Janeiro, Maracanã, Rio de Janeiro 20550-013, Brazil; thiprubart@hotmail.com

**Keywords:** histoplasmosis, *Histoplasma capsulatum*, endemic mycoses, COVID-19, diagnosis

## Abstract

The acute form of histoplasmosis usually occurs after the exposition of more than one individual to a common environmental source harboring *Histoplasma capsulatum*. Here, we present two cases of acute pulmonary histoplasmosis seen within two weeks at a reference center for infectious diseases at Rio de Janeiro, Brazil. The patients did not present a common epidemiologic history for histoplasmosis, however both presented COVID-19 before the onset of histoplasmosis symptoms. Due to the difficulties in the diagnosis of acute histoplasmosis, novel laboratory methods such as Western Blot and PCR were included in the investigation of these cases. Both patients presented negative cultures for *H. capsulatum* and negative urinary galactomannan. However, they presented H and M bands in the Western blot as well as a positive *H. capsulatum* DNA detection in sputum. These results were available approximately 36 h after sample collection, fastening the beginning of treatment of one patient. Both patients progressed well with itraconazole treatment. These cases suggest that COVID-19 may facilitate the development of acute pulmonary histoplasmosis and, therefore, clinicians must be aware of this differential diagnosis in patients from endemic areas with fever and coughing after recovery from COVID-19.

## 1. Introduction

Histoplasmosis is a global systemic mycosis highly endemic in certain regions of the Americas, including Brazil. It is caused by the dimorphic fungus *Histoplasma capsulatum* that predominately occurs in soil containing large amounts of bird or bat droppings. The infection occurs through the inhalation of fungal microconidia after perturbation of these environmental sources [1]. The disease is usually associated with immunosuppressive conditions, mostly aids, clinically presenting severe acute disseminated forms. Underlying lung disorders can predispose to chronic pulmonary histoplasmosis, whereas acute and subacute pulmonary forms mainly occur in healthy individuals after a large fungal inoculum inhalation [1,2]. These clinical forms are less known, often misdiagnosed as bacterial pneumonia and pulmonary tuberculosis.

Fungal tropical diseases have been neglected over the years, and the financial support for their diagnosis, treatment, and research is much lower than those available for other infectious diseases causing similar mortality [3]. This scenario is expected to get worse in the context of the Coronavirus Disease 2019 (COVID-19) pandemic [4]. Severe acute respiratory syndrome (SARS) related to this viral disease is known to increase the risk of invasive fungal infections [5,6]. In addition, patients suffering from endemic mycoses and COVID-19 coinfection seem to be an at-risk population and have a poor prognosis [7]. Thus far, histoplasmosis associated with COVID-19 has been reported in aids-related cases [8,9,10].

The authors aim to report two cases of acute pulmonary histoplasmosis following COVID-19, emphasizing the importance of considering endemic mycoses as differential diagnoses in respiratory syndromes and the need to improve the arsenal of diagnostic tools for endemic mycoses. Finally, the authors raise a question: Can COVID-19 represent a risk for pulmonary fungal infections?

## 2. Materials and Methods

### 2.1. Case Reports

#### 2.1.1. Case 1

**Figure 1:**
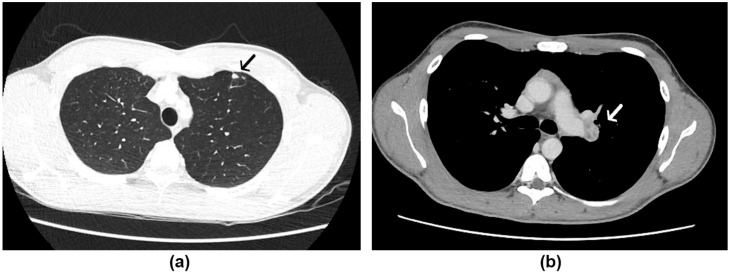
Chest tomography of case 1: (**a**) Nodular opacities on the periphery of the anterior segment of the upper left lobe (black arrow), (**b**) mediastinal lymph nodes (white arrow).

A 20-year-old male patient was admitted to the Evandro Chagas National Institute of Infectious Diseases (INI/Fiocruz), reporting high fever (39 °C) in the 1st week of February 2021. He had been followed by a pulmonologist due to a persistent dry cough after presenting a mild case of COVID-19 (confirmed through a positive real-time reverse-transcription PCR [RT-PCR] of a nasopharyngeal swab) 4 months before. The patient reported a great dust exposure while playing soccer on a ground field with bird excreta 5 weeks before the fever presented. The initial investigation included a chest tomography, which revealed small nodular opacities without calcifications, measuring up to 0.8 cm, on the periphery of the anterior segment of the upper left lobe, associated with some confluent vessels, as well as left pulmonary hilum enlargement, and small mediastinal lymph nodes (Figure 1).

Laboratory analyses (hematological and biochemistry) were within normal ranges, except for a high erythrocyte sedimentation rate (ESR)—34 mm (normal range <15 mm). Anti-HIV serology was negative. As fever started, levofloxacin was introduced, but no improvement occurred after 48 h of regular antibiotic use. A new RT-PCR of the nasopharyngeal swab was collected, which was negative. Serology for histoplasmosis was included in the investigation and, as the M band in double immunodiffusion test was present, itraconazole 200 mg/day was prescribed, and the patient was referred to our institute, a reference center for endemic mycoses in Rio de Janeiro, Brazil, where more diagnostic tests for histoplasmosis were performed.

#### 2.1.2. Case 2

**Figure 2:**
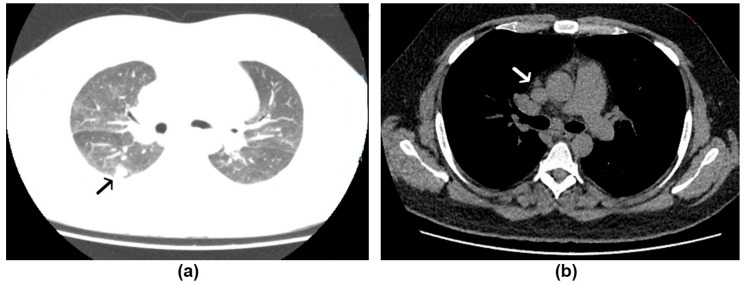
Chest tomography of case 2: (**a**) An irregular nodule (12 mm × 11 mm) of soft tissue density surrounded by ground-glass opacity in the superior segment of the right inferior lobe (black arrow); (**b**) lymph node enlargement (up to 21 mm × 16 mm) in the mediastinum and right pulmonary hilum (white arrow).

In the 3rd week of February 2021, a 32-year-old male patient, an intensive care physician from the reference hospital for severe COVID-19 at our institute, reported a 10-day fever reaching 39 °C, associated with dry cough, headache, and asthenia. These symptoms began a month after he was discharged from hospitalization to treat a bronchiolitis obliterans organizing pneumonia (BOOP) related to a severe COVID-19 confirmed by RT-PCR. During hospitalization, the patient received a corticosteroid pulse with methylprednisolone for BOOP therapy and progressed well. After the onset of these new symptoms, he was submitted to laboratory analyses, including a new RT-PCR of the nasopharyngeal swab to detect SARS-CoV-2, which was negative. Hematological and biochemistry analyses did not present noteworthy alterations. Anti-HIV serology was negative. Tuberculosis was ruled out (negative GeneXpert^®^ MTB/RIF Ultra from the sputum). A chest tomography showed tenuous ground-glass opacities in almost 25% of the lungs, suggesting clearance of the previous inflammatory viral pneumonia, along with a small irregular nodule in the superior segment of the right inferior lobe and mediastinal lymph nodes (Figure 2). The patient did not report any risk-activity for pulmonary mycoses but reported the presence of many bats near his residence in the Western part of Rio de Janeiro city.

As both patients presented a general good clinical condition at admission, they remained on outpatient follow-up after clinical samples collection (induced sputum, serum, and urine) for laboratory tests as detailed below. Itraconazole 200 mg/day was maintained for case 1, and he was guided to complete 3 months of treatment. As case 2 was more symptomatic, itraconazole was introduced at a higher dose (400 mg/day for 8 days), followed by 200 mg/day for 3 months. Both patients showed good adherence to the treatment and progressed well.

### 2.2. Mycological Examination

After preparation, direct examination of the sputum was done with 10% KOH and Giemsa stain. Cultures were performed on modified Sabouraud dextrose agar and mycosel agar (both from BD, Sparks, MD, USA) at 25 °C up to 30 days. Two slant tubes of each medium were used to improve the odds of *H. capsulatum* isolation. If putative Histoplasma colonies grew, they would be subcultured in ML-Gema agar medium [11], for 7 to 14 days at 37 °C for dimorphism confirmation.

### 2.3. DNA Extraction and Nested Polymerase Chain Reaction for H. capsulatum

DNA from the respiratory samples was extracted using the QIAamp DNA Mini Kit (QIAGEN^®^, Hilden, Germany), according to the manufacturer’s instructions. As controls, we used induced sputum samples from a patient with proven histoplasmosis (positive control) and from a patient with COVID-19 and without histoplasmosis (negative control). The efficacy of DNA extraction was estimated using the Nanovue^TM^ Plus Spectophotrometer (GE Healthcare, Buckinghamshire, UK). *H. capsulatum* nested PCR aimed the 100 kDa protein. Two sets of primers, (HCI 5′- GCG TTC CGA GCC TTC CAC CTC AAC—3′; HCII 5′- ATG TCC CAT CGG GCG CCG TGT AGT—3′; HCIII 5′- GAG ATC TAG TCG CGG CCA GGT TCA—3′; HCIV 5′- AGG AGA GAA CTG TAT CGG TGG CTT G-3′) (Invitrogen, Thermo Fisher Scientific, Lithuania) were used as previously described [12], with minor modifications. The outer and inner sets amplified 290 and 210 bp fragments, respectively. The 1st reaction consisted of 10 µL DNA in a total volume of 50 µL with final concentrations of 10 mM Tris-HCl (pH 8.3), 50 mM KCl, 2.5 mM MgCl_2_, 1.5 U of Taq DNA polymerase (Invitrogen), 1 µM of each outer primer, and 100 µM of each deoxynucleoside triphosphate (Roche Diagnostics, Basiland, Switzerland). The mix for the nested PCR was similar, except that 2 µL of the product from the 1st PCR, 50 mM each deoxynucleoside triphosphate, and 1 µM each inner primer set were used. The first PCR was cycled once at 94 °C for 5 min; 35 times at 94 °C for 30 s, 65 °C for 30 s, and 72 °C for 1 min; and then once at 72 °C for 5 min. For the 2nd amplification, the reaction mixture was thermally cycled once at 94 °C for 5 min, 30 times at 94 °C for 30 s and 72 °C for 1 min, and then once at 72 °C for 5 min. As additional controls, DNA extracted from the ATCC 26,032 (G-217B) *H. capsulatum* strain and a reaction without any DNA template (water control) were included in the assays. Bands around the expected size (210 bp) were excised from the agarose gel, purified using the illustraTM GFXTM PCR DNA and Gel Band Purification Kit (GE Healthcare, Buckinghamshire, UK) according to the manufacturer instructions, and sequenced at the sequencing platform (PDTIS/Fiocruz) at Oswaldo Cruz Foundation, which uses the ABI 3730xl- Applied Biosystems machine and the BigDye Terminator v3.1 cycle sequencing kit (Thermo Fisher Scientific, Waltham, MA, USA). Primers HcIII and HcIV were used for forward and reverse DNA sequencing. Sequences from both DNA strands were edited with the Sequencher version 4.6 software package (Gene Codes Corporation, Ann Arbor, MI, USA), and aligned using the Mega version 7.0 software. Sequences were deposited at the NCBI GenBank database. Homology analysis of these sequences were performed in GenBank.

### 2.4. Antibody Detection by Double Immunodiffusion (DID) and Western Blot (WB)

The DID test was performed following the methodology described by Ouchterlony [13] using the crude histoplasmin antigen [14], while WB followed a validated methodology described by our group [15] using the purified and deglycosylated histoplasmin antigen obtained according to a previously established protocol [16]. Briefly, the antigen/antibody precipitation reaction in the DID occurred in a 1% agarose gel supported by a glass slide. Ten microliters of antigen (in the central well) and patient sera (peripheral wells) were loaded individually. The slides were incubated in a humid chamber at room temperature for 48 h for diffusion and formation of precipitins. Then, they were washed with 5% sodium citrate for 2 h at room temperature and subsequently in 0.9% NaCl solution for 46 h at room temperature. Gels were dried and stained in 0.5% Coomassie brilliant blue R (Sigma Chemical Co., St. Louis, MO, USA) in an acetic acid-ethanol-water mixture as solvent (1:4.5:4.5). Excess dye was removed in an acetic acid-methanol-water mixture as solvent (1:4:5). The reaction was considered positive when a precipitation identity line was formed between antigen and antibody wells. For WB, proteins were resolved by SDS-PAGE followed by electrotransfer to nitrocellulose membranes. The membranes were incubated with patient’s sera diluted 1:100, and posteriorly goat anti-human immunoglobulin G—alkaline phosphatase conjugates (Jackson ImmunoResearch, EUA) (1:3000 dilution). The signal was detected using a substrate solution consisting of 5-bromo-4-chloro-3-indolylphosphate (BCIP) and nitroblue tetrazolium (NBT). After color development, strips were rinsed exhaustively in deionized water. Bands with 115 and 88 kDa were recorded as positive for H and M antigens of *H. capsulatum*, respectively. As controls, serum samples from a patient with proven histoplasmosis (positive control), from a patient with COVID-19 and without *H. capsulatum* infection (negative control), and from a healthy person (normal human serum control) were also tested. An additional negative control consisted of a secondary antibody control, that was, without the addition of any serum sample.

### 2.5. Galactomannan Enzyme Immunoassays (EIA)

The antigen detection tests were performed on urine and serum samples from the 2 patients herein described and the positive and negative control patients described above. The urine samples were used in the immunoassay clarus Histoplasma Galactomannan EIA [HGM201] performed according to the manufacturer’s instructions (IMMY, Norman, OK, USA). Although the manufacturer’s instructions describe the use of this kit only for urine samples, for research purposes, we also tested serum samples of the patients following the established protocol for urine. The serum samples were also tested for galactomannan reactivity by the Platelia™ Aspergillus EIA (BioRad Laboratories, Hercules, CA, USA), which was directed to be used with human serum samples, according to the manufacturer directions. Interpretation of the results was performed strictly according to the manufacturer’s instructions.

## 3. Results

### 3.1. Traditional Methods of Diagnosis

Direct examination (10% KOH) of sputum samples from both cases was performed on the same day of sample collection, but they did not reveal fungal structures compatible with agents of pulmonary mycoses. In addition, the Giemsa stain did not reveal yeast-like cells compatible with *H. capsulatum*. Similarly, cultures from both cases were also negative: Case 1 yielded only colonies of non-pathogenic fungi, and case 2 did not present any fungal growth after 30 days of incubation.

Antibody detection by DID was positive in the patient from case 1 (titer 1:32), who presented the M band, and negative in the patient from case 2.

### 3.2. Novel Methods of Diagnosis

**Figure 3:**
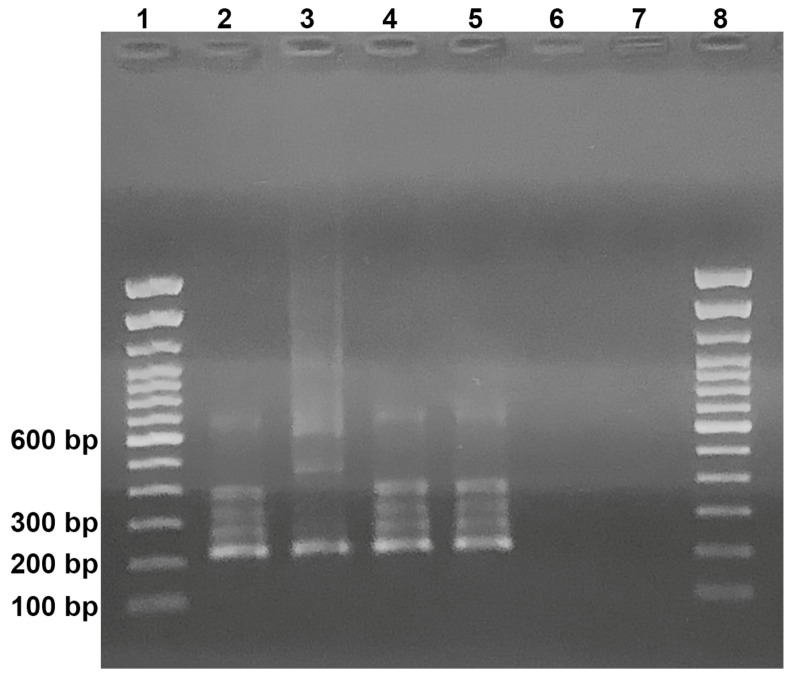
Nested Polymerase chain reaction for *H. capsulatum*: Slot 1 and 8 = molecular weight (100 bp DNA ladder— ThermoFisher Scientific, Inc.), slot 2 = case 1, slot 3 = case 2, slot 4 = positive control (patient with proven histoplasmosis), slot 5 = positive control (G217B DNA), slot 6 = negative control (patient with COVID-19), slot 7 = negative (water) control. The base pairs (bp) of representative bands are indicated at the left.

Nested PCR results were obtained on the day after the sample collection. Both patients yielded a 210 bp fragment compatible with the amplification product of the control *H. capsulatum* ATCC 26,032 (G-217B) strain (Figure 3). The 210 bp fragments were sequenced and 100% homology with the 100 kDa protein of *H. capsulatum* was observed. Sequences were deposited at the NCBI GenBank database under the numbers MW911367 (case 1) and MW911368 (case 2).

**Figure 4:**
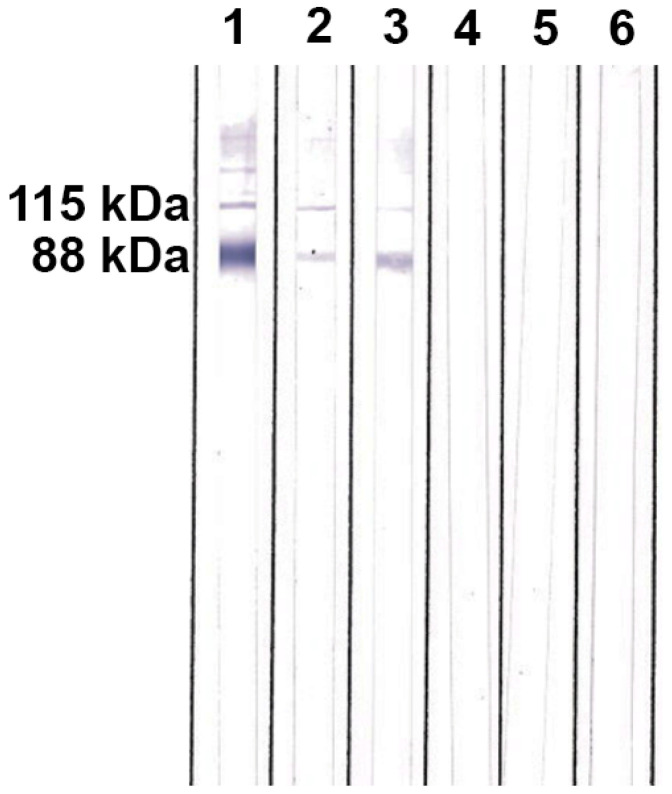
Western blot assay for anti-*Histoplasma* antibody detection: Line 1 = positive control (patient with proven histoplasmosis), line 2 = case 1, line 3 = case 2, line 4 = negative control (patient with COVID-19), line 5 = negative control (normal human serum), line 6 = secondary antibody control. Molecular weights of H and M antigens of *H. capsulatum* are indicated at the left.

The WB assay results were available on the same day of sample collection. Both patients yielded bands (115 and 88 kDa) corresponding to the H and M antigens of *H. capsulatum*, respectively (Figure 4).

*Histoplasma* antigen detection in the urine of the patient described as case 1 was negative. On the other hand, case 2 yielded a positive result. Moreover, galactomannan detection in sera was negative in the clarus Histoplasma Galactomanan EIA (IMMY) but positive in the Platelia^TM^ Aspergillus EIA. A summary of these results is presented in Table 1.

## 4. Discussion

When immunocompetent individuals get into contact with *H. capsulatum*, most remain asymptomatic or develop self-limited symptoms that usually resolve in a few days [1]. However, some individuals can develop pulmonary manifestations, and, even in this population, histoplasmosis can be a life-threatening disease [17]. This acute pulmonary form of histoplasmosis usually occurs in small outbreaks, when more than one individual in a defined period and community suddenly present with symptoms [18]. These outbreaks are usually related to a common environmental source, and Rio de Janeiro city is a known hot spot of histoplasmosis outbreaks in Brazil [19]. We were not able to detect a common environmental source or risk-activity for the two patients herein described, but both had a history of recent SARS-CoV-2 infection.

It has been described that COVID-19 can trigger some diseases in recovered patients. Multiple sclerosis was described in a patient six months after a COVID-19 episode due to a virus-induced neuroimmunopathologic condition [20]. Some children develop a multisystem inflammatory syndrome possibly triggered by molecular mimicry or by a dysregulated immune response following SARS-CoV-2 infection [21]. Here, we present pulmonary histoplasmosis as a potential complication in patients recovered from COVID-19. We envisage a few hypotheses to explain this association.

First, the pulmonary inflammation induced by SARS-CoV-2 infection and/or COVID-19 related lung damage might facilitate that patients develop acute histoplasmosis after inhalation of infective conidia in the environment. This is likely what happened with the patient reported as case 1, who had contact with a potential environmental *H. capsulatum* source after recovering from COVID-19. Even presenting mild symptoms, it has been described that patients with COVID-19 can have severe lung injuries without developing dyspnea or respiratory distress [22].

Second, the COVID-19 associated pulmonary damage or the corticosteroid therapy used in the management of severe COVID-19 cases may reactivate latent *H. capsulatum* foci inside the lungs. This is likely what could have happened with the patient from case 2, who probably had already been previously infected by this fungal agent as he lives in an area with bats, the major animal involved in histoplasmosis transmission and dissemination [23,24]. However, the remarkable presence of lymph node enlargement suggests primary disease.

Diagnosis of acute pulmonary histoplasmosis is a challenge. Most clinicians are not aware of symptoms, which are nonspecific. Culture does not have good sensitivity, and antibodies are usually detected by classic DID only in convalescent sera [2,25]. Antigen detection has an elevated sensitivity (around 83%) in acute histoplasmosis, but some patients present negative results, especially if only urine is tested [2,26]. In agreement with the literature, cultures from both patients were negative, and only one patient was positive in the DID test. The use of a validated Western blot assay [15] to enhance detection of antibodies against *H. capsulatum* H and M antigens and a molecular-based diagnostic test [26] to detect *H. capsulatum* DNA directly from sputum samples confirmed the diagnosis in both cases. The results of these tests were available within 36 h of sample collection and were fundamental to support itraconazole treatment in the patient from case 2. Both started recovering from histoplasmosis symptoms after two weeks of antifungal therapy.

Antigen detection in histoplasmosis is largely used in disseminated forms of the disease in immunosuppressed patients [27]. It has been described that, for better performance in patients with acute histoplasmosis, serum and urine should be tested together [28], which guided us to test both samples from each patient. Unfortunately, the specific *Histoplasma* antigen detection assay (clarus Histoplasma Galactomannan EIA, IMMY) is validated only for urine samples [29]. The Platelia^TM^ Aspergillus EIA is a test developed for the detection of the *Aspergillus* galactomannan antigen in human serum or bronchoalveolar lavage. However, some studies suggest that this assay may be helpful as an adjunctive test for the diagnosis of histoplasmosis, due to the serological cross-reactivity between these two fungi [30,31]. In the present study, one patient had a positive urinary *Histoplasma* antigen test, which is compatible with the report that around 64% of patients with acute pulmonary histoplasmosis present antigenuria [28]. Both patients presented detectable galactomannan levels only in the Platelia^TM^ Aspergillus assay. They did not present any symptom compatible with aspergillosis, and neutrophil counts were within normal ranges, which indicates that the positivity in this test was due to the cross-reactivities previously described. Moreover, the results indicate that the clarus Histoplasma Galactomannan EIA is indeed not recommended to screen seric levels of *H. capsulatum* antigens.

Chest tomography scans of both patients were suggestive of histoplasmosis, which may have facilitated the clinical suspicion and diagnosis of these two patients. Since Rio de Janeiro is an endemic area for histoplasmosis [32] and one of the most cities affected by SARS-CoV-2 in Brazil [33], we suppose that the two cases herein presented are the tip of the iceberg of the real magnitude of this problem. Clinicians should be aware of this life-threatening mycosis in patients with fever and coughing recovered from COVID-19, regardless of the clinical presentations of the viral infection. An active search of similar cases is necessary to confirm if COVID-19 facilitates the onset of histoplasmosis in endemic areas. The combination of novel and sensitive diagnostic methods based in antigen, antibody, and DNA detection should help in this task.

## Figures and Tables

**Table 1:** Galactomannan results of the two patients with acute pulmonary histoplasmosis following COVID-19.

Case	Urine (clarus, IMMY ^1^)	Serum (clarus, IMMY)	Serum (Platelia, Bio-Rad ^2^)
1	0.46	0.39	0.72
2	1.38	0.33	0.56
PC ^3^	37.64	35.98	1.21
NC ^4^	0.13	0.28	0.25

^1^ Results present as EIA units. Samples with EIA units ≥1.00 are considered positive; ^2^ Results present as index. Samples with an index ≥0.50 are considered positive; ^3^ PC: Positive control (patient with proven histoplasmosis); ^4^ NC: Negative control (patient with COVID-19).

## Data Availability

The data presented in this study are available on request from the corresponding author. The data are not publicly available due to privacy and ethical reasons.
